# The Dipeptides Ile-Tyr and Ser-Tyr Exert Distinct Effects on Catecholamine Metabolism in the Mouse Brainstem

**DOI:** 10.1155/2016/6020786

**Published:** 2016-02-11

**Authors:** Kazuki Moriyasu, Takashi Ichinose, Akane Nakahata, Mitsuru Tanaka, Toshiro Matsui, Shigeki Furuya

**Affiliations:** ^1^Laboratory of Functional Genomics and Metabolism, Graduate School of Bioresource and Bioenvironmental Sciences, Kyushu University, Fukuoka 812-8581, Japan; ^2^Laboratory of Food Analysis, Graduate School of Bioresource and Bioenvironmental Sciences, Kyushu University, Fukuoka 812-8581, Japan; ^3^Innovative Bio-Architecture Center, Kyushu University, Fukuoka 812-8581, Japan

## Abstract

Catecholamine synthesis and transmission in the brain are influenced by the availability of Tyr in the body. In this study, we compared the effects of oral administration of Tyr-containing dipeptides Ile-Tyr, Ser-Tyr, and Tyr-Pro with Tyr alone on catecholamine metabolism in the mouse brainstem. Among these dipeptides, Ile-Tyr administration led to increases in dopamine, the dopamine metabolites homovanillic acid, and 3,4-dihydroxyphenylacetic acid, compared to administration of Ser-Tyr, Tyr-Pro, or Tyr alone. In comparison, administration of Ser-Tyr induced significantly increasing noradrenaline turnover, while Tyr-Pro administration suppressed dopamine turnover. Therefore, oral administration of Ile-Tyr, Ser-Tyr, and Tyr-Pro differentially affected metabolism of dopamine and noradrenaline. These observations strongly suggest that Tyr-containing dipeptides exert distinct effects on catecholamine metabolism in the brainstem when ingested orally.

## 1. Introduction

Catecholamines are known to be critical to key brain functions such as cognition, emotion, executive function, mood, and spatial and working memory and to immune system homeostasis in the brain [1, 2]. Degeneration of central catecholaminergic systems could therefore contribute to a variety of psychiatric and neurological diseases that are associated with cognitive and emotional alterations and dysregulation of neuroinflammatory responses. Indeed, diminishment of central noradrenaline (NA, also known as norepinephrine) and dopamine (DA) transmission have been implicated in the pathophysiology of Parkinson's and Alzheimer's diseases [3], as well as major depressive disorder [4, 5].

In addition to Tyr derived from proteins in food,* de novo* synthesis of Tyr from Phe by phenylalanine hydroxylase (EC 1.14.16.1) occurs in the liver. Circulating Tyr enters the brain via amino acid transporters in the blood-brain barrier and serves as the amino acid precursor of the catecholamine neurotransmitters DA, NA, and adrenaline. Hence, catecholamine synthesis and transmission in the brain are influenced by the availability of Tyr in the body [6–8].

Our recent studies demonstrated that among eight dipeptide sequences containing the Tyr residue present frequently in soybean proteins of glycinin, *β*-conglycinin *α* subunit, and *β*-conglycinin *β* subunit, synthetic Ile-Tyr (IY), Ser-Tyr (SY), and Tyr-Pro (YP) had higher apparent permeability in human intestinal epithelial Caco-2 cell monolayers than other five dipeptides [9]. When administrated orally to mice, these three dipeptides increased serum Tyr concentrations more than Tyr alone [9, 10]. Furthermore, oral ingestion of SY, compared to an equivalent dose of IY, YP, or Tyr alone, led to an efficient and significant rise in free Tyr levels and stimulates NA metabolism in the cerebral cortex and hippocampus of adult mice [9]. These observations indicate that Tyr-containing dipeptides act differentially on the central noradrenergic system. Since NA and DA are synthesized in neurons of the locus coeruleus and substantia nigra, respectively, these findings raise the possibility that oral ingestion of SY and other Tyr-containing dipeptides derived from food proteins may modulate catecholamine metabolism in brainstem nuclei as we previously reported in forebrain regions [9]. The objective of the present study is to investigate the effects of orally ingested Tyr-containing dipeptides on catecholamine metabolism in the mouse brainstem. We show that orally ingested IY and SY dipeptides exert distinct effects on the metabolism of catecholamines in the brainstem.

## 2. Materials and Methods

### 2.1. Materials

Dopamine, 3,4-dihydroxyphenylacetic acid (DOPAC), homovanillic acid (HVA), 3-methoxytyramine (3-MT), noradrenaline, 3-methoxy-4-hydroxyphenylethyleneglycol (MHPG), normetanephrine (NM), and l-Tyr were obtained from Sigma-Aldrich (St. Louis, MO, USA). The dipeptides IY, SY, and Tyr-Pro (YP) were synthesized using a 9-fluorenylmethoxycarbonyl (Fmoc) solid-phase synthesis method according to the manufacturer's instructions (Kokusan Chemical Co., Osaka, Japan) [9].

### 2.2. Animal Experiments

All animal experiments were performed in accordance with the Standards Relating to the Care and Management of Laboratory Animals and Relief of Pain (notice number 88, Ministry of the Environment, Government of Japan). Experiments were approved by the Animal Ethics Committee of Kyushu University (permit number: A25-121). C57BL/6N mice at 8 weeks of age were purchased from Charles River Laboratories Japan, Inc. (Kanagawa, Japan). Mice were maintained in a pathogen-free animal facility (25 ± 1°C) under a 12-hour light/dark cycle for 2 weeks with free access to water and standard laboratory chow (CE-2; CLEA Japan Inc., Tokyo, Japan). They were then transferred to a breeding room in the laboratory, with a controlled environment as described above, and kept with water and standard laboratory chow for 2 days. Mice were then sorted into four groups. Each group was given IY, SY, YP, or Tyr in saline (50 *μ*mol/mL/50 g body weight) via oral gavage. Mice were sacrificed 30 min after dosing. The brainstems were isolated on an ice-cooled plastic plate, weighed, frozen in liquid N_2_, and stored at −80°C.

### 2.3. Measurement of Catecholamine and Metabolite Levels

Levels of catecholamines and their metabolites in the brainstem were determined using high-performance liquid chromatography with electrochemical detection (HPLC-ECD), as described previously [9]. Reversed-phase chromatographic analysis was performed using the HTEC-500 system (Eicom, Kyoto, Japan) with a reversed-phase column (Eicompack SC-5ODS, 3 mm *φ*  × 150 mm; Eicom) and a graphite working electrode (EICOM WE-3G; 12 mm *φ*) with an Ag/AgCl reference electrode. The potential of the working electrode was set at +750 mV. The acetate-citrate buffer (pH 3.5) used for separation (flow rate: 0.5 mL/min at 25°C) contained 0.053 M citric acid, 0.047 M sodium acetate, 5 mg/L EDTA, 193 mg/L sodium octyl sulfonate (Nacalai Tesque, Inc., Kyoto, Japan), and 17% methanol (v/v).

### 2.4. Statistical Analysis

Results are expressed as mean ± SEM. Differences between groups were analyzed with one-way ANOVA followed by Tukey-Kramer's test for* post hoc* analysis. *p* values < 0.05 were considered significant. All statistical tests were conducted using KaleidaGraph, version 4.0 (Synergy Software, Reading, PA, USA).

## 3. Results and Discussion

**Figure 1:**
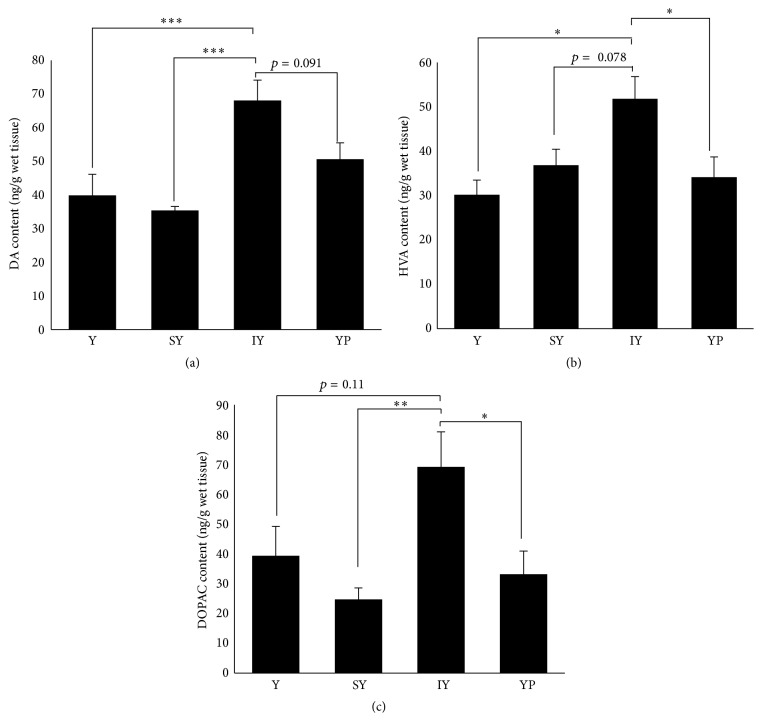
Effects of dipeptide ingestion on DA, HVA, and DOPAC levels in the brainstem. DA (a) and HVA (b) concentrations in the brainstem were determined 30 min after oral ingestion of dipeptides or Tyr alone. Data are expressed as mean ± SEM (*n* = 5 for Y-, IY-, and YP-groups; *n* = 6 for SY-group). Comparisons between Tyr- and dipeptide-groups were analyzed using one-way ANOVA followed by Tukey-Kramer's test for* post hoc* analysis. ^*∗*^ *p* < 0.05, ^*∗∗*^ *p* < 0.01, and ^*∗∗∗*^ *p* < 0.005.

Brainstem DA metabolism was affected by administration of IY. DA content in the brainstem of IY-group increased significantly to 170% and 192% levels of those of Tyr- and SY-groups, respectively (Figure 1(a)). There was a nonsignificant trend toward increase in DA content of IY-group over YP-group. Furthermore, IY administration resulted in a significant increase in HVA content, compared to administration of Tyr alone or YP (Figure 1(b)). DOPAC content in IY-group also showed a significant increase (versus SY- or YP-group) and a trend toward increase over Tyr-group (Figure 1(c)). Neither SY nor YP administration caused increases in DA and its metabolite levels over Y-group.

**Figure 2:**
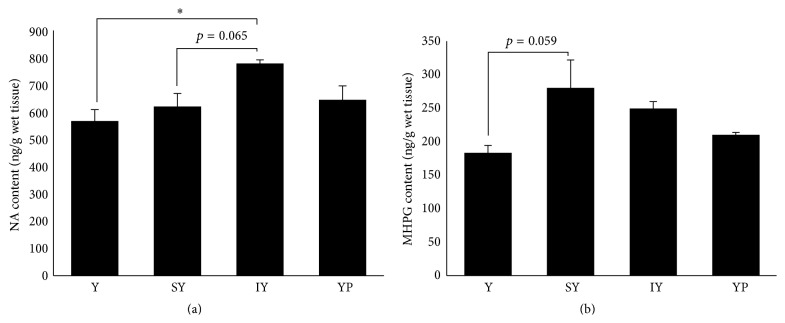
Effects of dipeptide ingestion on NA and MHPG levels in the brainstem. NA (a) and MHPG (b) concentrations in the brainstem were determined 30 min after oral ingestion of dipeptides or Tyr alone. Data are expressed as mean ± SEM (*n* = 5 for Tyr-, IY-, and YP-groups; *n* = 6 for SY-group). Comparisons between Tyr- and dipeptide-groups were analyzed using one-way ANOVA followed by Tukey-Kramer's test for* post hoc* analysis. ^*∗*^ *p* < 0.05.

Unlike DA metabolism, the dipeptide administration led to subtle changes in NA metabolism of the brainstem. NA content of IY-group exhibited a significant 137% increase versus Tyr-group and a trend toward increasing level over SY-group (Figure 2(a)), while MHPG content did not alter significantly by these treatments (Figure 2(b)). The administration of SY showed significant and marked increase in MHPG content of the cerebral cortex and hippocampus [9], whereas a nonsignificant but markedly increasing MHPG content was observed in SY-group, compared to Tyr-group (153%, *p* = 0.059).

**Figure 3:**
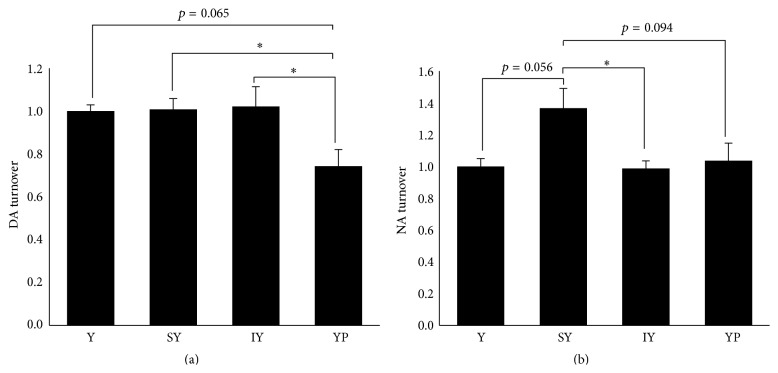
Effects of dipeptide ingestion on turnover of DA and NA in the brainstem. DA and NA turnover in the brainstem were evaluated by determining the concentration ratio of (HVA + DOPAC) to DA and that of MHPG to NA, respectively. Data are expressed as the normalized value to the mean of Tyr-group (*n* = 5 for Tyr-, IY-, and YP-groups; *n* = 6 for SY-group). Comparisons between groups were analyzed using one-way ANOVA followed by Tukey-Kramer's test for* post hoc* analysis. ^*∗*^ *p* < 0.05.

We then quantified the turnover of DA and NA in the brainstem by determining the ratios of DOPAC plus HVA to DA and of MHPG to NA. As shown in Figure 3(a), YP administration resulted in a significant decrease in DA turnover (versus SY- or IY-group). Although DA turnover was not enhanced by IY administration, this is probably because IY increased DA level as well as DOPAC and HVA (Figure 1). SY administration caused a significant (versus IY) and nonsignificant (versus Tyr- or YP-group) stimulation of NA turnover (Figure 3(b)). It is of note that stimulation of NA turnover by SY administration has been observed also in the cerebral cortex and hippocampus [9].

IY, SY, and YP dipeptide motifs are common in the major soy proteins glycinin and *α* and *β* subunits of *β*-conglycinin [9]. Among these three dipeptides and five other synthetic Tyr-containing dipeptides whose sequences occur in soy proteins, we demonstrated that IY exhibited the highest apparent permeability coefficient in an intestinal transport model consisting of human intestinal Caco-2 cell monolayers [9]. Thus, IY may have a unique affinity for membrane peptide transporters that facilitate absorption from the intestinal lumen. This seems a likely mechanism by which IY administration leads to increase in both DA and NA levels in the brainstem. However, further study will be necessary to determine the underlying mechanism by which IY and SY exert different effects on the catecholamine metabolism in the brainstem and forebrain regions.

The loss of dopaminergic neurons in the substantia nigra associated with decreased levels of DA is a pathological hallmark of Parkinson's disease, while Alzheimer's disease exhibits neuronal degeneration in several subcortical nuclei. Postmortem human brain studies reported that noradrenergic neurons originating exclusively from the locus coeruleus in brainstem are degenerated in brains of both Parkinson's disease and Alzheimer's disease patients, which are often accompanied by decreased levels of NA also [3, 11, 12]. There is ample experimental evidence that NA is neuroprotective and loss of noradrenergic neurons in the locus coeruleus sensitizes neurons to damage in these diseases [13, 14]. Of particular interest, animal studies have shown that NA exerts anti-inflammatory action on microglia cells via suppression of their inflammatory gene expression, thereby preventing central neurons from long-term neurotoxic insults such as deposition of *β*-amyloid peptides [15, 16]. Thus, development of effective and safe nutraceutical interventions contributing to maintaining central NA as well as DA may be relevant for prevention or delay in the onset of these diseases.

A recent study using 2,4,6-trinitrobenzene sulfonate derivatization-aided LC-TOF-MS detected IY and SY in a commercially available soy protein hydrolysate composed mainly of di- and tripeptides [17]. The concentrations of IY and SY were estimated to be 2211 ± 133 and 2188 ± 199 *μ*g/g of hydrolysate, respectively. Since ingestion of IY and SY can lead to enhanced DA synthesis and NA turnover, respectively, it will be interesting to investigate whether chronic ingestion of these dipeptides and/or soy protein hydrolysates may mitigate neuropathological symptoms of Parkinson's disease and/or Alzheimer's disease models.

## 4. Conclusions

In conclusion, our present study demonstrates for the first time that oral intake of IY, SY, and YP elicits distinct effects on the metabolism of DA and NA in the brainstem. Although there is little information regarding the mechanism underlying the modulatory effects of these peptides on the brainstem metabolism of DA and NA, it seems likely that different transport and stability properties in body fluids may confer different influences on catecholamine metabolism. The present study may offer a new opportunity for the future development of Tyr-containing dipeptides as an effective food material beneficial for brain function.

## Abbreviations

DA: Dopamine
DOPAC: 3,4-Dihydroxyphenylacetic acid
HVA: Homovanillic acid
3-MT: 3-Methoxytyramine
MHPG: 3-Methoxy-4-hydroxyphenylethyleneglycol
NA: Noradrenaline
NM: Normetanephrine.
